# Poly[(μ_3_-5-*tert*-butyl­benzene-1,3-di­carboxyl­ato)di­pyridine­cobalt(II)]

**DOI:** 10.1107/S1600536813026640

**Published:** 2013-10-02

**Authors:** Kyungkyou Noh, Jaheon Kim

**Affiliations:** a Department of Chemistry, Soongsil University, 369 Sangdo-Ro, Dongjak-Gu, Seoul 156-743, Republic of Korea

## Abstract

In the title compound, [Co(C_12_H_12_O_4_)(C_5_H_5_N)_2_]_*n*_, the Co^II^ cation is coordinated by four O atoms from three 5-*tert*-butyl­benzene-1,3-di­carboxyl­ate anions and two N atoms from pyridine mol­ecules in a distorted octa­hedral geometry. One carboxyl­ate group of the anionic ligand chelates a Co^II^ cation while another carboxyl­ate group bridges two Co^II^ cations, resulting in a polymeric layer parallel to (101). Weak C—H⋯O hydrogen bonds occur between adjacent polymeric layers. In the crystal, one of pyridine mol­ecules is equally disordered over two positions.

## Related literature

For metal-organic frameworks composed of cobalt cations and 5-*tert*-butyl­benzene-1,3-di­carboxyl­ate ligands without additional bridging ligands, see: Chen *et al.* (2011 ▶); Du *et al.* (2009 ▶); Ma *et al.* (2009 ▶); Qin & Ju (2010 ▶). For a copper(II) complex with 5-*tert*-butylbenzene-1,3-­dicarboxyl­ate ligand, see: Li & Zhou (2010 ▶).
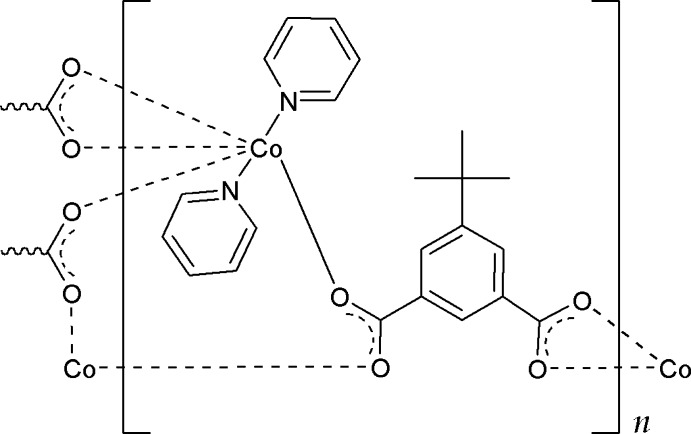


## Experimental

### 

#### Crystal data

[Co(C_12_H_12_O_4_)(C_5_H_5_N)_2_]
*M* *_r_* = 437.34
Monoclinic, 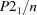

*a* = 9.7357 (3) Å
*b* = 15.6699 (6) Å
*c* = 13.0764 (5) Å
β = 94.791 (1)°
*V* = 1987.93 (12) Å^3^
*Z* = 4
Mo *K*α radiation
μ = 0.90 mm^−1^
*T* = 173 K
0.50 × 0.40 × 0.30 mm

#### Data collection

Bruker SMART APEX CCD diffractometer
Absorption correction: multi-scan (*SADABS*; Bruker, 2001 ▶) *T*_min_ = 0.663, *T*_max_ = 0.775
12370 measured reflections
4580 independent reflections
3843 reflections with *I* > 2σ(*I*)
*R*_int_ = 0.021

#### Refinement

*R*[*F* ^2^ > 2σ(*F* ^2^)] = 0.049
*wR*(*F* ^2^) = 0.135
*S* = 1.11
4580 reflections
314 parameters
66 restraints
H-atom parameters constrained
Δρ_max_ = 1.25 e Å^−3^
Δρ_min_ = −0.64 e Å^−3^

### 

Data collection: *SMART* (Bruker, 2007 ▶); cell refinement: *SAINT* (Bruker, 2007 ▶); data reduction: *SAINT*; program(s) used to solve structure: *SHELXTL* (Sheldrick, 2008 ▶); program(s) used to refine structure: *SHELXTL*; molecular graphics: *CrystalMaker* (CrystalMaker Software, 2013 ▶); software used to prepare material for publication: *publCIF* (Westrip, 2010 ▶).

## Supplementary Material

Crystal structure: contains datablock(s) global, I. DOI: 10.1107/S1600536813026640/xu5741sup1.cif

Structure factors: contains datablock(s) I. DOI: 10.1107/S1600536813026640/xu5741Isup2.hkl

Additional supplementary materials:  crystallographic information; 3D view; checkCIF report

## Figures and Tables

**Table 1:** Selected bond lengths (Å)

Co1—O1	2.014 (2)
Co1—O2^i^	2.0608 (19)
Co1—O3^ii^	2.324 (2)
Co1—O4^ii^	2.103 (2)
Co1—N1	2.182 (3)
Co1—N2	2.195 (9)
Co1—N2*A*	2.117 (11)

Symmetry codes: (i) 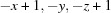; (ii) 
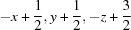
.

**Table 2:** Hydrogen-bond geometry (Å, °)

*D*—H⋯*A*	*D*—H	H⋯*A*	*D*⋯*A*	*D*—H⋯*A*
C14—H14⋯O4^iii^	0.95	2.55	3.287 (5)	134

Symmetry code: (iii) 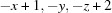.

**Fig. 1.**
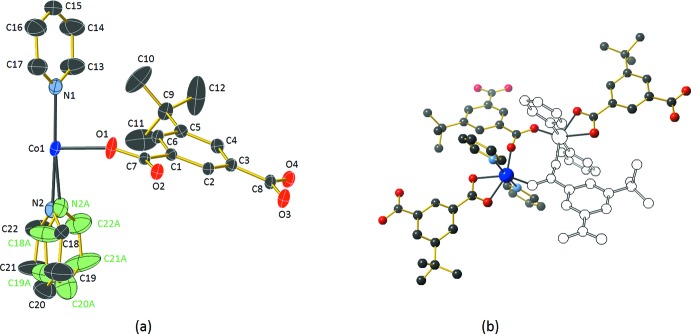
(a) An asymmetric unit of the title compound is shown with the atomic numbering scheme. Displacement ellipsoids are drawn at 50% probability level. Hydrogen atoms are omitted for simplicity. (b) The full coordination environment of the cobalt cluster is shown with ball-and-stick models. The atoms in the asymmetric unit are highlited with empty balls.

**Fig. 2.**
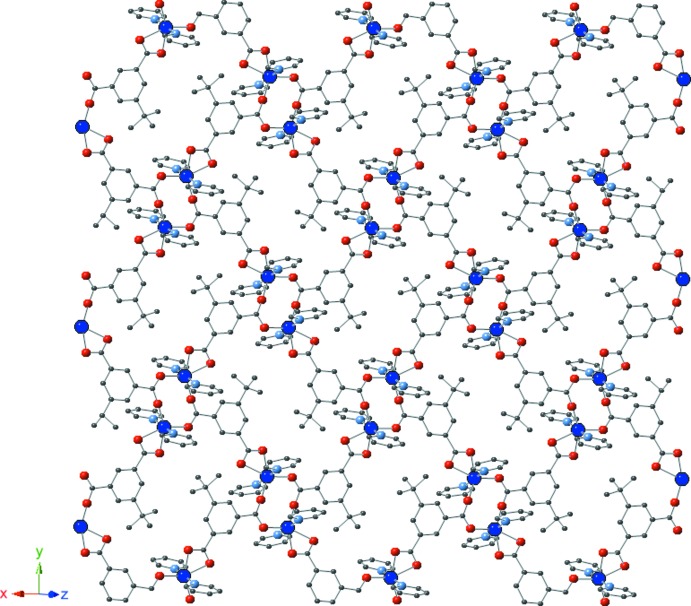
A two-dimensional layer approximately parallel to the (101) plane is represented.

**Fig. 3.**
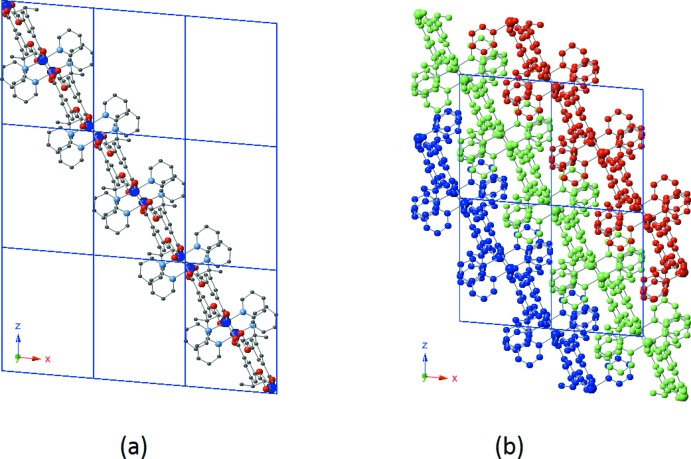
(a) The layer in Figure 2 is drawn with the lattice lines along the b axis. (b) A packing view is shown with three layers represented with different colors.

## supplementary crystallographic information

### 1. 1. Comment

The bent ligand, 5-*tert*-1,3-benzene­dicarb­oxy­lic acid (H_2_BDC_^t^Bu) can form inter­esting metal-organic polyhedra (MOPs) through solvothermal reactions with copper ions which frequently give paddle-wheel type clusters (Li & Zhou, 2010). In contrast, the reactions between cobalt ion and H_2_BDC_^t^Bu provide various types of metal-organic frameworks (MOFs) including even a molecular cyclic compound: for examples such as rings, chains, layers, and three-dimensional networks, see Chen *et al.*, 2011; Du *et al.*, 2009; Ma *et al.*, 2009; Qin & Ju, 2010. The combination of a diverse coordination property of cobalt ion, and a bulky tert-butyl groups in the anionic ligand can give a possibility of making inter­esting MOFs. For example, a reaction between cobalt ions and the ligands in polar solvent, the hydro­phobic tertiary butyl groups assembled together, making polar carboxyl­ate groups be exposed to pores to give hydro­philic environment (Ma *et al.*, 2009). In searching for new Co-BDC_^t^Bu frameworks, we obtained a two-dimensional layered structure of which inter-layer space is filled with hydro­phobic and coordinated pyridine molecules and *tert*-butyl groups of the anionic ligands.

### 2. 2. Experimental

Cobalt(II) nitrate tetra­hydrate (40 mg) and 5-*tert*-butyl­benzene-1,3-di­carb­oxy­lic acid (31 mg) were dissolved in a mixed solvent (*N*,*N*-di­methyl­formamide, 8.0 ml; ethanol, 0.4 ml; pyridine, 0.4 ml). This reaction mixture was transferred to a Teflon-lined vessel (23 ml), and heated at 378 K and for 2 days. Large violet block crystals were obtained and used for single crystal X-ray diffraction analyses.

### 2.1. 3. Refinement

In the asymmetric unit, one of two pyridine molecules is disordered over two positions with a site occupancy factor of 0.5, respectively. The site occupancy factors were converged to 0.50787 and 0.49213 for each disordered portion with isotropic thermal parameters, and thus the factors were fixed to 0.5 respectively at the final stage of refinement; the thermal parameters did not converge when the site occupancy factors were refined together. Due to the disorder, the geometry of disordered pyridine was restrained by FLAT and SAME SHELX instructions. In addition, the anisotropic thermal parameters of N2, N2A, C30, and C30A atoms were treated isotropically using an ISOR instruction. However, the C19A thermal parameters were equaled to those of C19 using an EADP instruction. Hydrogen atoms of the aromatic and methyl groups were placed at calculated positions with C—H = 0.95 Å and 0.98 Å, respectively and allowed to ride with U_iso_(H) = 1.2 U_eq_(C).

### Crystal data

**Table:**

[Co(C_12_H_12_O_4_)(C_5_H_5_N)_2_]	*F*(000) = 908
*M**_r_* = 437.34	*D*_x_ = 1.461 Mg m^−^^3^
Monoclinic, *P*2_1_/*n*	Mo *K*α radiation, λ = 0.71073 Å
Hall symbol: -P 2yn	Cell parameters from 6406 reflections
*a* = 9.7357 (3) Å	θ = 2.5–28.2°
*b* = 15.6699 (6) Å	µ = 0.90 mm^−^^1^
*c* = 13.0764 (5) Å	*T* = 173 K
β = 94.791 (1)°	Block, violet
*V* = 1987.93 (12) Å^3^	0.50 × 0.40 × 0.30 mm
*Z* = 4	

### Data collection

**Table:**

Bruker SMART APEX CCD diffractometer	4580 independent reflections
Radiation source: fine-focus sealed tube	3843 reflections with *I* > 2σ(*I*)
Graphite monochromator	*R*_int_ = 0.021
phi and ω scans	θ_max_ = 27.9°, θ_min_ = 2.0°
Absorption correction: multi-scan (*SADABS*; Bruker, 2001)	*h* = −12→12
*T*_min_ = 0.663, *T*_max_ = 0.775	*k* = −17→20
12370 measured reflections	*l* = −17→13

### Refinement

**Table:**

Refinement on *F*^2^	66 restraints
Least-squares matrix: full	H-atom parameters constrained
*R*[*F*^2^ > 2σ(*F*^2^)] = 0.049	*w* = 1/[σ^2^(*F*_o_^2^) + (0.059*P*)^2^ + 3.5707*P*] where *P* = (*F*_o_^2^ + 2*F*_c_^2^)/3
*wR*(*F*^2^) = 0.135	(Δ/σ)_max_ = 0.001
*S* = 1.11	Δρ_max_ = 1.25 e Å^−^^3^
4580 reflections	Δρ_min_ = −0.64 e Å^−^^3^
314 parameters	

### Special details

**Table:**

Geometry. Geometry. All e.s.d.'s (except the e.s.d. in the dihedral angle between two l.s. planes) are estimated using the full covariance matrix. The cell e.s.d.'s are taken into account individually in the estimation of e.s.d.'s in distances, angles and torsion angles; correlations between e.s.d.'s in cell parameters are only used when they are defined by crystal symmetry. An approximate (isotropic) treatment of cell e.s.d.'s is used for estimating e.s.d.'s involving l.s. planes.Refinement. Refinement of F^2^ against ALL reflections. The weighted *R*-factor *wR* and goodness of fit S are based on F^2^, conventional *R*-factors *R* are based on F, with F set to zero for negative F^2^. The threshold expression of F^2^ > 2sigma(F^2^) is used only for calculating *R*-factors(gt) *etc*. and is not relevant to the choice of reflections for refinement. *R*-factors based on F^2^ are statistically about twice as large as those based on F, and R– factors based on ALL data will be even larger.

### Fractional atomic coordinates and isotropic or equivalent isotropic displacement parameters (Å^2^)

**Table:**

	*x*	*y*	*z*	*U*_iso_*/*U*_eq_	Occ. (<1)
Co1	0.44382 (4)	0.12814 (2)	0.54417 (3)	0.02396 (13)	
O1	0.4499 (3)	0.01260 (13)	0.61177 (17)	0.0394 (5)	
O2	0.4464 (2)	−0.12667 (11)	0.58392 (15)	0.0263 (4)	
O3	0.1852 (2)	−0.30891 (12)	0.83448 (16)	0.0342 (5)	
O4	0.1101 (2)	−0.24206 (13)	0.96689 (15)	0.0305 (4)	
C1	0.3528 (3)	−0.07399 (16)	0.73420 (19)	0.0207 (5)	
C2	0.3038 (3)	−0.15329 (16)	0.76379 (19)	0.0210 (5)	
H2	0.3153	−0.2024	0.7228	0.025*	
C3	0.2379 (3)	−0.15960 (17)	0.8543 (2)	0.0234 (5)	
C4	0.2262 (3)	−0.08775 (18)	0.9164 (2)	0.0261 (5)	
H4	0.1828	−0.0932	0.9785	0.031*	
C5	0.2770 (3)	−0.00814 (17)	0.8893 (2)	0.0244 (5)	
C6	0.3373 (3)	−0.00254 (16)	0.7962 (2)	0.0236 (5)	
H6	0.3687	0.0514	0.7744	0.028*	
C7	0.4206 (3)	−0.06185 (16)	0.63520 (19)	0.0203 (5)	
C8	0.1748 (3)	−0.24228 (17)	0.8864 (2)	0.0257 (5)	
C9	0.2610 (3)	0.07170 (19)	0.9557 (2)	0.0326 (6)	
C10	0.3841 (5)	0.1310 (3)	0.9510 (4)	0.0771 (17)	
H10A	0.3773	0.1777	1.0001	0.116*	
H10B	0.3850	0.1544	0.8816	0.116*	
H10C	0.4694	0.0990	0.9683	0.116*	
C11	0.1368 (5)	0.1215 (3)	0.9076 (5)	0.0868 (19)	
H11A	0.0537	0.0863	0.9085	0.130*	
H11B	0.1520	0.1362	0.8365	0.130*	
H11C	0.1252	0.1739	0.9469	0.130*	
C12	0.2442 (10)	0.0500 (3)	1.0652 (3)	0.127 (3)	
H12A	0.3234	0.0162	1.0930	0.190*	
H12B	0.1595	0.0168	1.0693	0.190*	
H12C	0.2386	0.1026	1.1051	0.190*	
N1	0.6310 (3)	0.16214 (15)	0.63878 (19)	0.0302 (5)	
C13	0.6260 (4)	0.1716 (3)	0.7395 (3)	0.0621 (12)	
H13	0.5418	0.1585	0.7678	0.074*	
C14	0.7352 (5)	0.1993 (4)	0.8058 (3)	0.0688 (13)	
H14	0.7255	0.2045	0.8772	0.083*	
C15	0.8549 (4)	0.2186 (3)	0.7673 (3)	0.0533 (10)	
H15	0.9311	0.2387	0.8108	0.064*	
C16	0.8649 (5)	0.2088 (4)	0.6645 (4)	0.0734 (15)	
H16	0.9488	0.2211	0.6353	0.088*	
C17	0.7502 (4)	0.1806 (3)	0.6033 (3)	0.0550 (10)	
H17	0.7583	0.1743	0.5318	0.066*	
N2	0.2472 (9)	0.1037 (5)	0.4542 (7)	0.0300 (18)	0.5
C18	0.2420 (11)	0.0415 (5)	0.3860 (6)	0.055 (3)	0.5
H18	0.3222	0.0071	0.3851	0.066*	0.5
C19	0.1310 (12)	0.0203 (7)	0.3143 (8)	0.091 (3)	0.5
H19	0.1370	−0.0260	0.2679	0.109*	0.5
C20	0.0168 (10)	0.0680 (5)	0.3141 (7)	0.067 (2)	0.5
H20	−0.0607	0.0562	0.2671	0.080*	0.5
C21	0.0130 (8)	0.1349 (5)	0.3834 (7)	0.082 (4)	0.5
H21	−0.0664	0.1699	0.3855	0.098*	0.5
C22	0.1299 (8)	0.1485 (5)	0.4496 (7)	0.068 (3)	0.5
H22	0.1262	0.1945	0.4967	0.082*	0.5
N2A	0.2579 (10)	0.0868 (5)	0.4638 (8)	0.038 (2)	0.5
C18A	0.2377 (10)	0.0736 (5)	0.3620 (8)	0.074 (4)	0.5
H18A	0.3156	0.0820	0.3239	0.089*	0.5
C19A	0.1159 (12)	0.0489 (5)	0.3055 (10)	0.091 (3)	0.5
H19A	0.1105	0.0410	0.2332	0.109*	0.5
C20A	0.0078 (12)	0.0374 (6)	0.3605 (8)	0.079 (3)	0.5
H20A	−0.0778	0.0206	0.3263	0.094*	0.5
C21A	0.0159 (9)	0.0488 (6)	0.4613 (7)	0.109 (6)	0.5
H21A	−0.0621	0.0404	0.4991	0.131*	0.5
C22A	0.1429 (8)	0.0734 (5)	0.5102 (7)	0.072 (3)	0.5
H22A	0.1477	0.0811	0.5824	0.086*	0.5

### Atomic displacement parameters (Å^2^)

**Table:**

	*U*^11^	*U*^22^	*U*^33^	*U*^12^	*U*^13^	*U*^23^
Co1	0.0331 (2)	0.01763 (19)	0.0226 (2)	−0.00231 (14)	0.01078 (14)	−0.00066 (13)
O1	0.0653 (16)	0.0189 (10)	0.0373 (12)	−0.0033 (10)	0.0248 (11)	0.0037 (8)
O2	0.0347 (10)	0.0209 (9)	0.0248 (9)	−0.0011 (8)	0.0112 (8)	−0.0027 (7)
O3	0.0502 (13)	0.0207 (10)	0.0335 (11)	−0.0038 (9)	0.0152 (9)	0.0009 (8)
O4	0.0393 (11)	0.0279 (10)	0.0258 (10)	−0.0057 (8)	0.0123 (8)	0.0022 (8)
C1	0.0210 (11)	0.0217 (12)	0.0195 (11)	0.0009 (9)	0.0027 (9)	0.0011 (9)
C2	0.0230 (12)	0.0188 (11)	0.0213 (12)	0.0006 (9)	0.0034 (10)	−0.0010 (9)
C3	0.0245 (12)	0.0228 (12)	0.0234 (13)	−0.0011 (10)	0.0039 (10)	0.0021 (10)
C4	0.0285 (13)	0.0279 (14)	0.0226 (13)	0.0004 (11)	0.0070 (10)	0.0005 (10)
C5	0.0267 (13)	0.0238 (13)	0.0229 (12)	0.0016 (10)	0.0031 (10)	−0.0027 (10)
C6	0.0271 (13)	0.0197 (12)	0.0242 (13)	−0.0006 (10)	0.0035 (10)	0.0008 (10)
C7	0.0199 (11)	0.0198 (12)	0.0213 (12)	0.0009 (9)	0.0035 (9)	0.0011 (9)
C8	0.0302 (14)	0.0231 (13)	0.0243 (13)	−0.0022 (10)	0.0046 (11)	0.0044 (10)
C9	0.0412 (17)	0.0268 (14)	0.0315 (15)	−0.0020 (12)	0.0128 (12)	−0.0084 (11)
C10	0.079 (3)	0.058 (3)	0.100 (4)	−0.031 (2)	0.045 (3)	−0.053 (3)
C11	0.067 (3)	0.070 (3)	0.121 (5)	0.031 (2)	−0.005 (3)	−0.051 (3)
C12	0.307 (11)	0.044 (3)	0.036 (2)	−0.032 (4)	0.056 (4)	−0.018 (2)
N1	0.0379 (13)	0.0244 (11)	0.0284 (12)	−0.0031 (10)	0.0042 (10)	0.0017 (9)
C13	0.052 (2)	0.102 (4)	0.0326 (19)	−0.022 (2)	0.0082 (16)	−0.013 (2)
C14	0.069 (3)	0.100 (4)	0.036 (2)	−0.016 (3)	−0.0039 (19)	−0.017 (2)
C15	0.056 (2)	0.048 (2)	0.052 (2)	−0.0120 (17)	−0.0178 (18)	0.0064 (17)
C16	0.048 (2)	0.112 (4)	0.059 (3)	−0.029 (3)	−0.005 (2)	0.024 (3)
C17	0.043 (2)	0.086 (3)	0.0362 (19)	−0.014 (2)	0.0033 (15)	0.0108 (19)
N2	0.023 (3)	0.025 (3)	0.042 (3)	0.007 (2)	0.006 (2)	0.002 (3)
C18	0.052 (5)	0.043 (4)	0.066 (7)	−0.020 (4)	−0.020 (5)	0.018 (4)
C19	0.054 (4)	0.151 (10)	0.065 (4)	−0.018 (5)	−0.006 (3)	0.000 (5)
C20	0.056 (4)	0.067 (4)	0.075 (4)	0.009 (3)	−0.015 (4)	−0.004 (4)
C21	0.040 (4)	0.076 (6)	0.123 (9)	0.027 (4)	−0.037 (5)	−0.056 (6)
C22	0.057 (5)	0.061 (5)	0.084 (7)	0.016 (4)	−0.008 (5)	−0.033 (5)
N2A	0.032 (4)	0.030 (4)	0.054 (4)	0.009 (3)	0.011 (3)	0.005 (3)
C18A	0.032 (4)	0.144 (13)	0.046 (5)	−0.005 (7)	0.003 (4)	−0.023 (8)
C19A	0.054 (4)	0.151 (10)	0.065 (4)	−0.018 (5)	−0.006 (3)	0.000 (5)
C20A	0.083 (5)	0.081 (5)	0.073 (5)	0.009 (4)	0.015 (4)	0.013 (4)
C21A	0.051 (5)	0.203 (16)	0.078 (7)	−0.065 (8)	0.030 (5)	−0.050 (9)
C22A	0.053 (5)	0.103 (8)	0.061 (5)	−0.022 (5)	0.016 (4)	−0.012 (5)

### Geometric parameters (Å, º)

**Table:**

Co1—O1	2.014 (2)	C12—H12B	0.9800
Co1—O2^i^	2.0608 (19)	C12—H12C	0.9800
Co1—O3^ii^	2.324 (2)	N1—C17	1.317 (4)
Co1—O4^ii^	2.103 (2)	N1—C13	1.330 (4)
Co1—N1	2.182 (3)	C13—C14	1.384 (5)
Co1—N2	2.195 (9)	C13—H13	0.9500
Co1—N2A	2.117 (11)	C14—C15	1.342 (6)
O1—C7	1.245 (3)	C14—H14	0.9500
O2—C7	1.254 (3)	C15—C16	1.365 (6)
O2—Co1^i^	2.0608 (19)	C15—H15	0.9500
O3—C8	1.254 (3)	C16—C17	1.391 (5)
O3—Co1^iii^	2.324 (2)	C16—H16	0.9500
O4—C8	1.271 (3)	C17—H17	0.9500
O4—Co1^iii^	2.102 (2)	N2—C22	1.337 (10)
C1—C6	1.398 (4)	N2—C18	1.320 (10)
C1—C2	1.397 (3)	C18—C19	1.410 (11)
C1—C7	1.513 (3)	C18—H18	0.9500
C2—C3	1.396 (4)	C19—C20	1.340 (12)
C2—H2	0.9500	C19—H19	0.9500
C3—C4	1.398 (4)	C20—C21	1.388 (10)
C3—C8	1.508 (4)	C20—H20	0.9500
C4—C5	1.398 (4)	C21—C22	1.388 (10)
C4—H4	0.9500	C21—H21	0.9500
C5—C6	1.399 (4)	C22—H22	0.9500
C5—C9	1.538 (4)	N2A—C22A	1.333 (11)
C6—H6	0.9500	N2A—C18A	1.346 (11)
C9—C12	1.494 (5)	C18A—C19A	1.398 (11)
C9—C10	1.522 (5)	C18A—H18A	0.9500
C9—C11	1.529 (6)	C19A—C20A	1.336 (12)
C10—H10A	0.9800	C19A—H19A	0.9500
C10—H10B	0.9800	C20A—C21A	1.325 (11)
C10—H10C	0.9800	C20A—H20A	0.9500
C11—H11A	0.9800	C21A—C22A	1.398 (10)
C11—H11B	0.9800	C21A—H21A	0.9500
C11—H11C	0.9800	C22A—H22A	0.9500
C12—H12A	0.9800		
			
O1—Co1—O2^i^	110.40 (8)	H11A—C11—H11C	109.5
O1—Co1—O4^ii^	153.91 (8)	H11B—C11—H11C	109.5
O2^i^—Co1—O4^ii^	95.64 (7)	C9—C12—H12A	109.5
O1—Co1—N2A	86.3 (2)	C9—C12—H12B	109.5
O2^i^—Co1—N2A	94.2 (3)	H12A—C12—H12B	109.5
O4^ii^—Co1—N2A	93.6 (2)	C9—C12—H12C	109.5
O1—Co1—N1	88.74 (10)	H12A—C12—H12C	109.5
O2^i^—Co1—N1	89.90 (9)	H12B—C12—H12C	109.5
O4^ii^—Co1—N1	89.70 (9)	C17—N1—C13	115.5 (3)
N2A—Co1—N1	174.4 (2)	C17—N1—Co1	124.9 (2)
O1—Co1—N2	94.1 (2)	C13—N1—Co1	119.4 (2)
O2^i^—Co1—N2	92.9 (2)	N1—C13—C14	124.4 (4)
O4^ii^—Co1—N2	86.0 (2)	N1—C13—H13	117.8
N1—Co1—N2	175.1 (2)	C14—C13—H13	117.8
O1—Co1—O3^ii^	94.57 (8)	C15—C14—C13	118.8 (4)
O2^i^—Co1—O3^ii^	154.98 (7)	C15—C14—H14	120.6
O4^ii^—Co1—O3^ii^	59.36 (7)	C13—C14—H14	120.6
N2A—Co1—O3^ii^	88.9 (3)	C14—C15—C16	118.7 (4)
N1—Co1—O3^ii^	89.01 (9)	C14—C15—H15	120.7
N2—Co1—O3^ii^	86.7 (2)	C16—C15—H15	120.7
C7—O1—Co1	162.0 (2)	C15—C16—C17	118.8 (4)
C7—O2—Co1^i^	125.77 (16)	C15—C16—H16	120.6
C8—O3—Co1^iii^	84.88 (16)	C17—C16—H16	120.6
C8—O4—Co1^iii^	94.51 (17)	N1—C17—C16	123.8 (4)
C6—C1—C2	119.7 (2)	N1—C17—H17	118.1
C6—C1—C7	118.1 (2)	C16—C17—H17	118.1
C2—C1—C7	122.1 (2)	C22—N2—C18	111.9 (8)
C3—C2—C1	119.3 (2)	C22—N2—Co1	129.5 (6)
C3—C2—H2	120.3	C18—N2—Co1	118.3 (7)
C1—C2—H2	120.3	N2—C18—C19	127.5 (10)
C2—C3—C4	120.0 (2)	N2—C18—H18	116.3
C2—C3—C8	121.6 (2)	C19—C18—H18	116.3
C4—C3—C8	118.4 (2)	C20—C19—C18	117.4 (11)
C5—C4—C3	121.6 (2)	C20—C19—H19	121.3
C5—C4—H4	119.2	C18—C19—H19	121.3
C3—C4—H4	119.2	C19—C20—C21	119.1 (9)
C4—C5—C6	117.4 (2)	C19—C20—H20	120.4
C4—C5—C9	121.9 (2)	C21—C20—H20	120.4
C6—C5—C9	120.7 (2)	C20—C21—C22	117.3 (7)
C1—C6—C5	121.9 (2)	C20—C21—H21	121.4
C1—C6—H6	119.1	C22—C21—H21	121.4
C5—C6—H6	119.1	N2—C22—C21	126.9 (8)
O1—C7—O2	124.5 (2)	N2—C22—H22	116.6
O1—C7—C1	117.0 (2)	C21—C22—H22	116.6
O2—C7—C1	118.5 (2)	C22A—N2A—C18A	111.5 (9)
O3—C8—O4	121.2 (2)	C22A—N2A—Co1	122.6 (8)
O3—C8—C3	120.7 (2)	C18A—N2A—Co1	125.9 (7)
O4—C8—C3	118.0 (2)	N2A—C18A—C19A	127.8 (9)
C12—C9—C10	109.1 (4)	N2A—C18A—H18A	116.1
C12—C9—C11	111.4 (5)	C19A—C18A—H18A	116.1
C10—C9—C11	105.7 (4)	C20A—C19A—C18A	115.1 (11)
C12—C9—C5	112.3 (3)	C20A—C19A—H19A	122.5
C10—C9—C5	110.9 (3)	C18A—C19A—H19A	122.5
C11—C9—C5	107.3 (3)	C19A—C20A—C21A	122.3 (11)
C9—C10—H10A	109.5	C19A—C20A—H20A	118.9
C9—C10—H10B	109.5	C21A—C20A—H20A	118.9
H10A—C10—H10B	109.5	C20A—C21A—C22A	117.9 (9)
C9—C10—H10C	109.5	C20A—C21A—C21A^iv^	115.4 (7)
H10A—C10—H10C	109.5	C20A—C21A—H21A	121.0
H10B—C10—H10C	109.5	C22A—C21A—H21A	121.0
C9—C11—H11A	109.5	N2A—C22A—C21A	125.4 (9)
C9—C11—H11B	109.5	N2A—C22A—H22A	117.3
H11A—C11—H11B	109.5	C21A—C22A—H22A	117.3
C9—C11—H11C	109.5		
			
C6—C1—C2—C3	1.1 (4)	C6—C5—C9—C12	−159.0 (5)
C7—C1—C2—C3	−177.7 (2)	C4—C5—C9—C10	146.6 (4)
C1—C2—C3—C4	−2.6 (4)	C6—C5—C9—C10	−36.7 (4)
C1—C2—C3—C8	175.9 (2)	C4—C5—C9—C11	−98.4 (4)
C2—C3—C4—C5	1.3 (4)	C6—C5—C9—C11	78.3 (4)
C8—C3—C4—C5	−177.3 (3)	C17—N1—C13—C14	0.4 (7)
C3—C4—C5—C6	1.4 (4)	Co1—N1—C13—C14	−174.9 (4)
C3—C4—C5—C9	178.2 (3)	N1—C13—C14—C15	0.3 (9)
C2—C1—C6—C5	1.7 (4)	C13—C14—C15—C16	−1.0 (8)
C7—C1—C6—C5	−179.4 (2)	C14—C15—C16—C17	1.0 (8)
C4—C5—C6—C1	−2.9 (4)	C13—N1—C17—C16	−0.4 (7)
C9—C5—C6—C1	−179.8 (3)	Co1—N1—C17—C16	174.5 (4)
Co1—O1—C7—O2	79.6 (7)	C15—C16—C17—N1	−0.3 (8)
Co1—O1—C7—C1	−102.2 (6)	C22—N2—C18—C19	−0.02 (6)
Co1^i^—O2—C7—O1	5.1 (4)	Co1—N2—C18—C19	−173.7 (5)
Co1^i^—O2—C7—C1	−172.99 (16)	N2—C18—C19—C20	0.03 (7)
C6—C1—C7—O1	−4.8 (4)	C18—C19—C20—C21	−0.09 (15)
C2—C1—C7—O1	174.1 (3)	C19—C20—C21—C22	0.14 (19)
C6—C1—C7—O2	173.5 (2)	C18—N2—C22—C21	0.08 (16)
C2—C1—C7—O2	−7.7 (4)	Co1—N2—C22—C21	172.8 (6)
Co1^iii^—O3—C8—O4	0.3 (3)	C20—C21—C22—N2	−0.1 (2)
Co1^iii^—O3—C8—C3	−179.1 (2)	C22A—N2A—C18A—C19A	0.04 (6)
Co1^iii^—O4—C8—O3	−0.3 (3)	Co1—N2A—C18A—C19A	−177.9 (5)
Co1^iii^—O4—C8—C3	179.0 (2)	N2A—C18A—C19A—C20A	−0.02 (7)
C2—C3—C8—O3	3.1 (4)	C18A—C19A—C20A—C21A	0.09 (15)
C4—C3—C8—O3	−178.4 (3)	C19A—C20A—C21A—C22A	−0.2 (2)
C2—C3—C8—O4	−176.3 (3)	C18A—N2A—C22A—C21A	−0.12 (15)
C4—C3—C8—O4	2.2 (4)	Co1—N2A—C22A—C21A	177.9 (5)
C4—C5—C9—C12	24.3 (5)	C20A—C21A—C22A—N2A	0.2 (2)

Symmetry codes: (i) −*x*+1, −*y*, −*z*+1; (ii) −*x*+1/2, *y*+1/2, −*z*+3/2; (iii) −*x*+1/2, *y*−1/2, −*z*+3/2; (iv) −*x*, −*y*, −*z*+1.

### Hydrogen-bond geometry (Å, º)

**Table:**

*D*—H···*A*	*D*—H	H···*A*	*D*···*A*	*D*—H···*A*
C14—H14···O4^v^	0.95	2.55	3.287 (5)	134

Symmetry code: (v) −*x*+1, −*y*, −*z*+2.
